# Identification of mRNA expression biomarkers associated with epilepsy and response to valproate with co-expression analysis

**DOI:** 10.3389/fneur.2022.1019121

**Published:** 2022-10-18

**Authors:** Jun Min, Qinglan Chen, Wenyue Wu, Jing Zhao, Xinming Luo

**Affiliations:** Department of Neurology, The Second Affiliated Hospital of Nanchang University, Nanchang, China

**Keywords:** epilepsy, valproate, drug resistance, WGCNA, T cells, biomarker

## Abstract

**Purpose:**

Valproate (VPA) resistance was reported to be an important predictor of intractable epilepsy. We conducted this study to identify candidate biomarkers in peripheral blood correlated with VPA resistance.

**Methods:**

The microarray dataset (GSE143272) was downloaded from the Gene Expression Omnibus database. Weighted gene co-expression network analysis (WGCNA) was performed to construct co-expression modules and obtain the most prominent module associated with VPA resistance. Differentially expressed genes (DEGs) between VPA-responsive and VPA-resistant patients were obtained using the “Limma” package in R. The intersections between the most prominent module and DEGs were identified as target genes. Metascape was performed to discover the possible involved pathways of the target genes. GeneCards database was used to know the function of each target gene.

**Results:**

All genes in the GSE143272 were divided into 24 different modules. Among these modules, the darkred module showed a pivotal correlation with VPA resistance. A total of 70 DEGs between VPA-responsive and VPA-resistant patients were identified. After taking the intersection, 25 target genes were obtained. The 25 target genes were significantly enriched in T cell receptor recognition, T cell receptor signaling pathway, regulation of T cell activation, cytokine–cytokine receptor interaction, and *in utero* embryonic development. Half of the target genes (*CD3D, CD3G, CXCR3, CXCR6, GATA3, GZMK, IL7R, LIME1, SIRPG, THEMIS, TRAT1*, and *ZNF683*) were directly involved in the T cell development, migration, and activation signaling pathway.

**Conclusion:**

We identified 25 target genes prominently associated with VPA resistance, which could be potential candidate biomarkers for epilepsy resistance in peripheral blood. The peripheral blood T cells may play a crucial role in VPA resistance. Those genes and pathways might become therapeutic targets with clinical usefulness in the future.

## Introduction

Epilepsy is characterized as a predisposition of the brain to generate epileptic seizures accompanied by neurobiological, cognitive, and psychosocial consequences (1). About 65 million people around the world suffer from epilepsy (2). One-third of patients with epilepsy fail to achieve sustained seizure freedom in spite of appropriately choosing and taking antiepileptic drugs (3). Pharmacoresistant epilepsy is associated with increased cognitive and psychiatric disorders, decreased quality of life, and even high risk of premature death (4, 5). The mechanisms of drug resistance in epilepsy remain elusive. Searching hematological diagnostic markers of drug resistance in epilepsy contributes to early diagnosis and treatment of refractory epilepsy.

Valproate (VPA) is the most commonly prescribed antiepileptic drug which is suitable for various epilepsy types including partial and generalized epilepsy (6). VPA can prevent seizures in 66% of patients with convulsive status epilepticus (7). However, almost one-third of patients are resistant to VPA treatment (7). Gesche et al. (8) revealed that VPA resistance is an important predictor of refractory idiopathic generalized epilepsy. The specificity and predictive value of VPA resistance in determining patients with epilepsy resistance is 100%, which is strongly associated with adverse social outcome and high seizure burden (8). Exploring the biomarker and mechanisms of VPA resistance is of great value for the study of epilepsy resistance.

Expression microarrays and high-throughput sequencing play an important role in promoting the research on drug resistance, identifying novel therapeutic targets, and accelerating drug discovery, and have been widely used in the study on drug resistance to chemotherapy and antibiotics (9–11). Previously, Wang et al. detected the differentially expressed genes (DEGs) in the peripheral blood of VPA-responsive and non-responsive pediatric patients with epilepsy and showed that specific cytokines and chemokines might participate in processes associated with VPA resistance (12). Compared with traditional expression profile analysis focusing on several DEGs, weighted gene co-expression network analysis (WGCNA) divides thousands of genes into dozens of gene modules with similar expression patterns and obtains the significant relationships between gene modules and specific phenotypes (13). WGCNA has been successfully applied to identify candidate biomarkers or therapeutic targets in various diseases and biological contexts (14–16).

In this study, we performed WGCNA analysis in peripheral blood expression profiles of patients with VPA-sensitive and VPA-resistant epilepsy and identified the mRNA expression biomarkers of VPA resistance. Our results revealed that the activation of peripheral blood T cells might have important pathophysiological significance in VPA resistance.

## Materials and methods

### Data collection

The microarray dataset (GSE143272) was downloaded from the Gene Expression Omnibus database (http://www.ncbi.nlm.nih.gov/geo/). This dataset consisted of 34 drug-naïve patients with epilepsy, 57 patients with different responses to antiepileptic drug monotherapy (phenytoin, VPA, and carbamazepine) during a follow-up, and 50 healthy control subjects. The peripheral blood samples were obtained and detected using an expression profiles array. In this article, only data from patients responsive and resistant to VPA were collected for further co-expression network analysis. In the GSE143272 dataset, patients on VPA therapy were followed-up over a period of 1 year for drug, dose, and serum drug concentrations and response to treatment after enrolment (17). During the course, patients who remained seizure-free were categorized as “VPA responsive” and who experienced at least 3 seizures were categorized as “VPA resistant” (17).

### Weighted gene co-expression network analysis

Before co-expression network construction, all samples were clustered to observe whether there were outliers. Two outlier samples (GSM4255896 and GSM4255845) were deleted. The WGCNA R package was used to construct a gene co-expression network. The expression matrix of all the genes was converted into an adjacency matrix, which was then converted into a topological dissimilarity matrix. To ensure that the weighted co-expression network conformed to the scale-free network in this process, a soft threshold of 7 was chosen. Then, a dynamic tree-cutting algorithm was used to cluster gene modules on the basis of topological matrix. The module-trait relationship was obtained by estimating the correlation between the module eigengene and the phenotype (gender, age, and VPA resistance). The module highly related to VPA resistance was selected. For each expression profile, gene significance was calculated as the absolute value of the correlation between expression profile and each trait; module membership was defined as the correlation of expression profile and each module eigengene. The significant module genes were denoted as genes with a *p*-value of gene significance in VPA resistance less than 0.05 and were used for subsequent analysis.

### Identification of DEGs

The “Limma” package in R was conducted to obtain DEGs between VPA-responsive and VPA-resistant patients, drug-naïve patients with epilepsy and healthy controls, and drug-naïve patients with epilepsy and patients taking VPA. If multiple probes matched the same gene, the mean signal intensity was computed. Genes with a *p*-value of < 0.05 and the |log2 FC| of > 0.5 were considered robust DEGs.

### Bio-function analysis

The Venn diagram was used to intersect the significant genes in the darkred module and the DEGs of VPA resistance obtained by Limma to identify target genes. The intersected genes were uploaded to Metascape (http://metascape.org/gp/index.html) to perform functional annotation analysis (18). GeneCards database (https://www.genecards.org/) was used to know the function of each target gene (19).

### Specificity verification of the target genes

To determine whether the target genes were related to VPA resistance rather than VPA or epilepsy, we intersected the target genes with the DEGs between drug-naïve patients with epilepsy and patients taking VPA. The same method was used to obtain the intersection of the target genes with DEGs between drug-naïve patients with epilepsy and healthy controls.

## Results

### Construction of weighted co-expression network and modules

**Figure 1:**
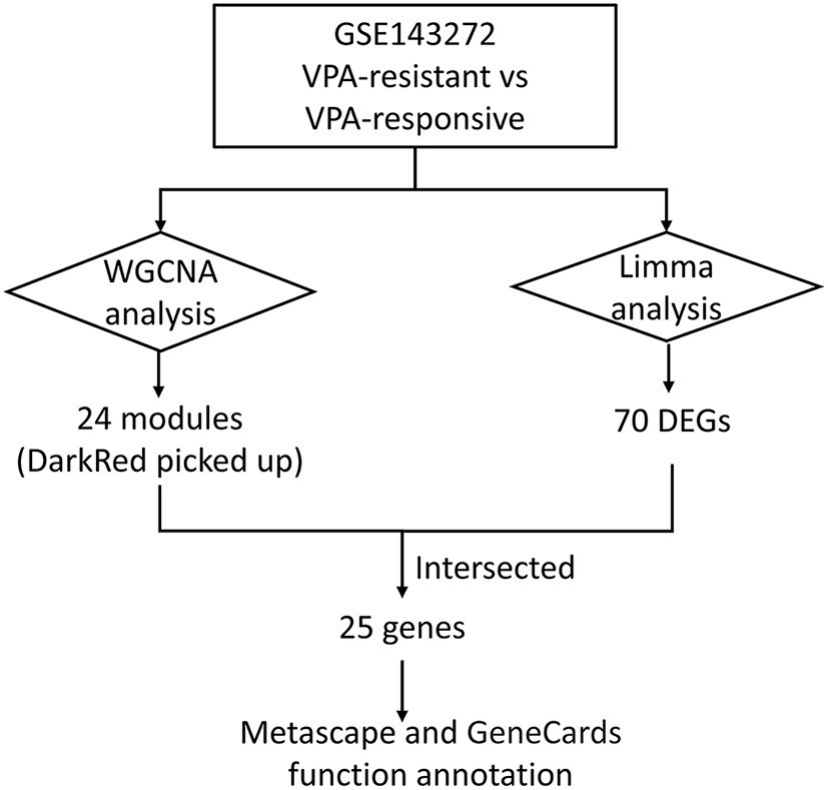
The workflow of this article.

**Figure 2:**
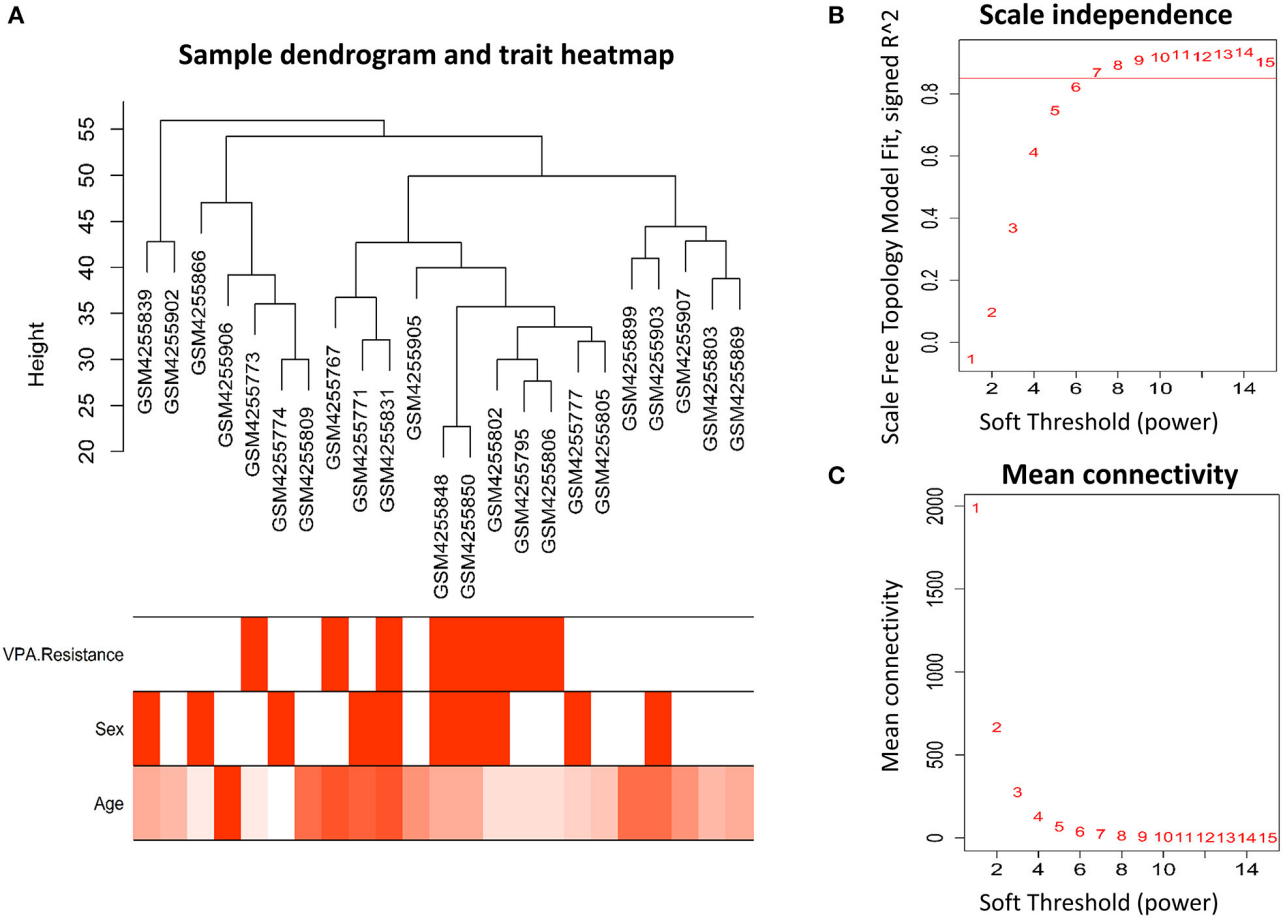
Clustering of samples and identification of soft-thresholding power. **(A)** The microarray dataset GSE143272 contained 9 VPA-resistant and 16 VPA-responsive samples. After clustering and removing two outliers, the remaining 23 samples were analyzed. The different color below denoted different disease status (VPA resistance, gender, and age). **(B)** The scale-independence and **(C)** mean connectivity for different soft threshold powers were calculated, and 7 was selected as the most fit power value to construct scale-free networks (VPA, valproate).

**Figure 3:**
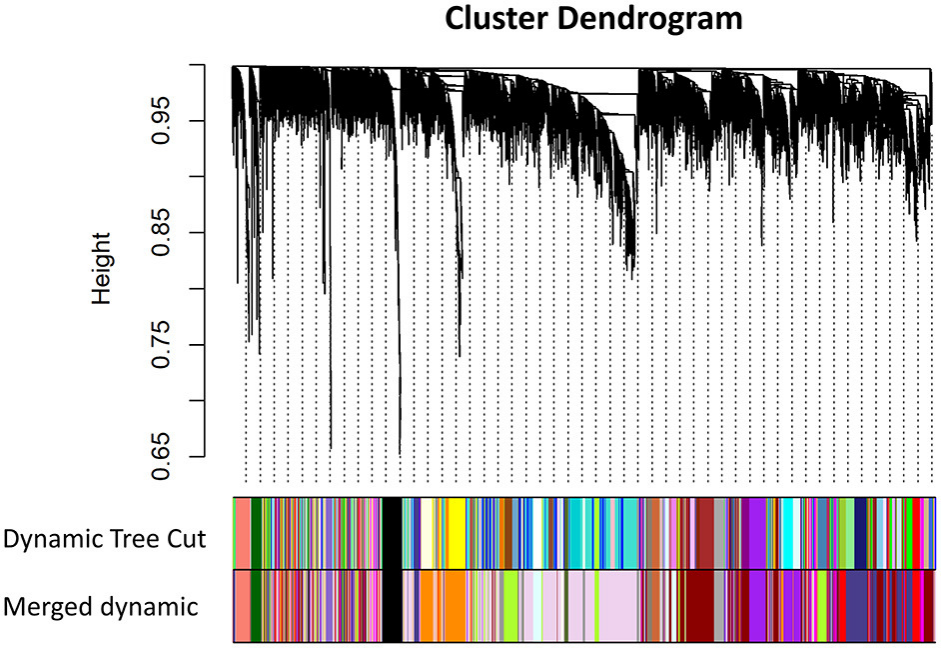
Cluster dendrogram of all genes and construction of co-expression modules by WGCNA in GSE143272. Each branch represented one gene, and every color bar below corresponded to one co-expression module. All genes were finally divided into 24 different modules after merging modules with high similarity (WGCNA, weighted gene co-expression network analysis).

The workflow of this article is shown in Figure 1. A total of 9 VPA-resistant and 16 VPA-responsive samples were included for co-expression network analysis. Two outlier samples (GSM4255896 and GSM4255845) were deleted after sample cluster analysis (Figure 2A). To conform to the scale-free network, the soft-thresholding power of 7 was selected to attain the balanced scale independence (Figure 2B) and mean connectivity (Figure 2C). All genes were hierarchically clustered using the dynamic hybrid tree cut method and the highly similar modules were merged. All genes were finally divided into 24 different modules according to the connectivity, as shown in Figure 3. The genes that could not be included in any modules were classified into the gray module, which was removed in the subsequent analysis.

### Correlation between modules and identification of key modules

**Figure 4:**
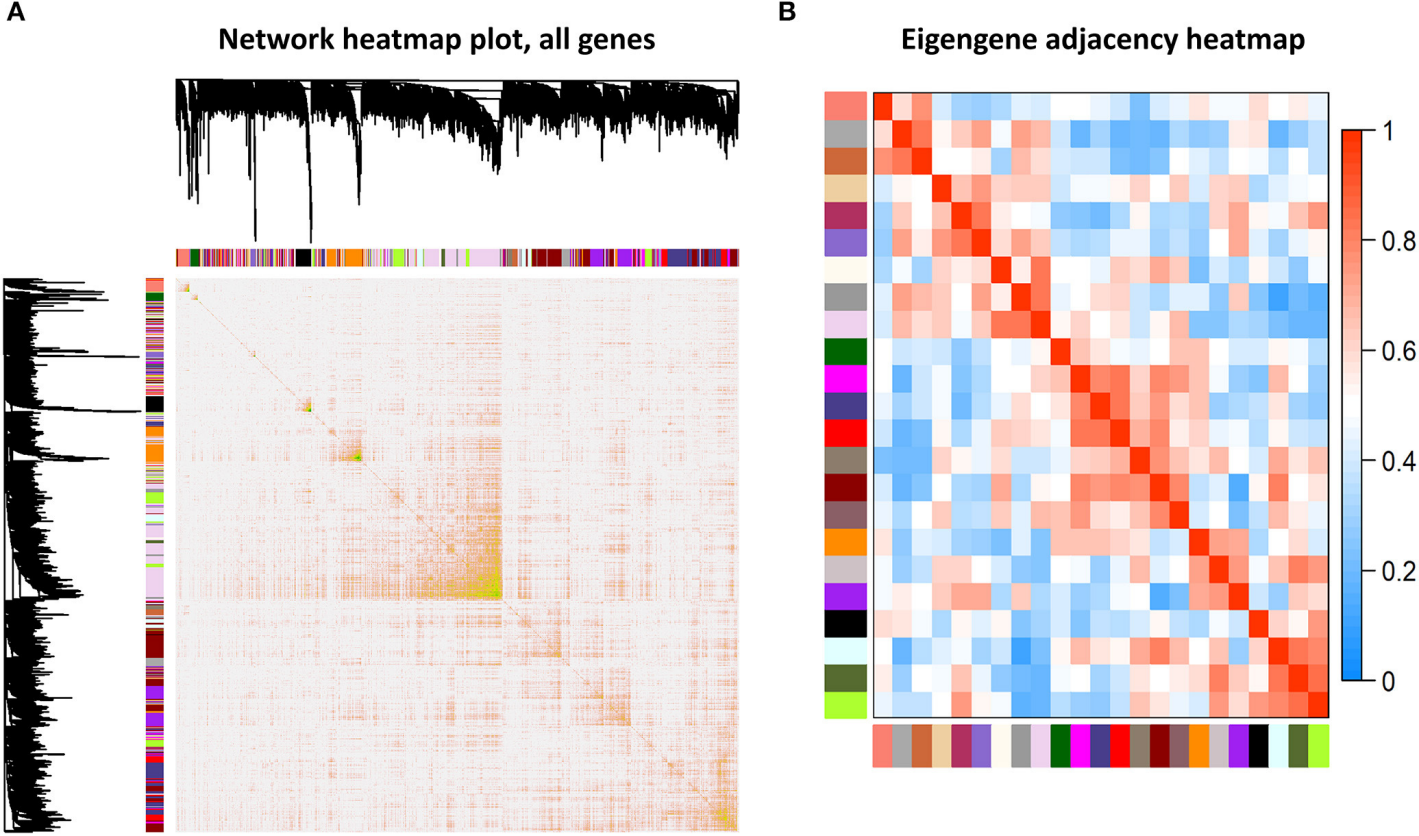
Association analysis of modules. **(A)** Different colored bars represented different modules. The depth of color represented the tightness of the connection among different modules. Significant dissimilarity in the connectivity existed among different modules. **(B)** Heat map to display the eigengene adjacencies in some representative modules.

**Figure 5:**
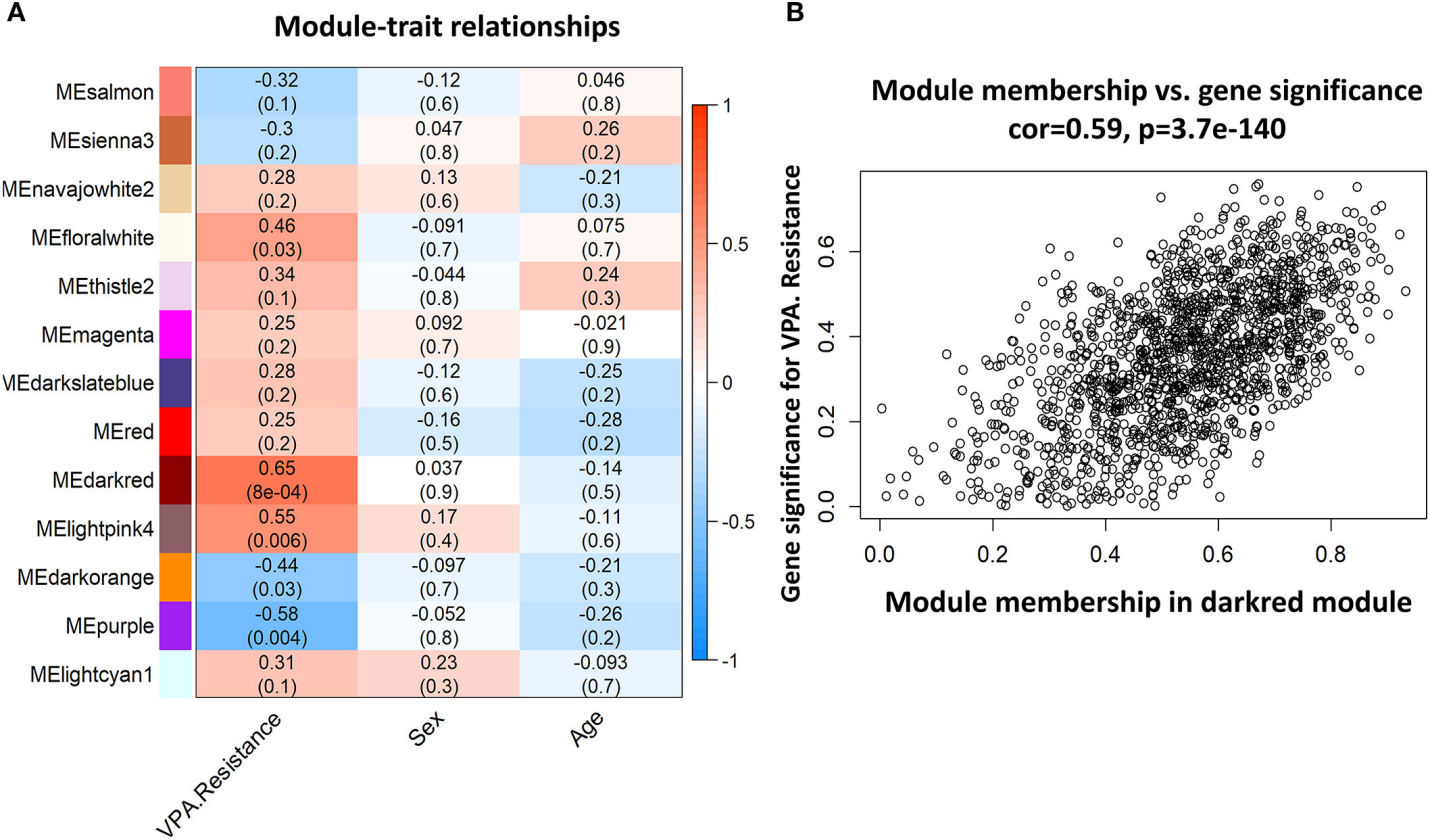
Representative heat map of modules-traits relationship and analysis of gene significance and module membership in the darkred module. **(A)** The heat map showed the relationship between different modules and each trait (VPA resistance, sex, or age). The number in each bar denoted the correlation coefficient and *p*-value. The darkred module is most related to VPA resistance. **(B)** Scatter diagram to show the gene significance in VPA resistance and module membership in the darkred module (VPA, valproate).

Among the 24 different modules, the interaction association (Figure 4A) and eigengene adjacency (Figure 4B) were analyzed and plotted. The results showed that modules were independent of each other, which indicated a high-degree independence of gene expression in different modules. Furthermore, we obtained modules related to VPA resistance, age, and gender through the module-traits correlation analysis. The results are shown in Figure 5A. The darkred, lightpink4, purple, darkorange, and floralwhite modules revealed a high correlation with disease type (responsive or resistant to VPA) compared with other modules (Figure 5A). None of the 24 modules showed a significant correlation with age and gender. The darkred module was the most significant module related to the VPA resistance. The gene significance and module membership in the darkred module were calculated, as shown in Figure 5B and Supplementary Table S1. In the darkred module, genes with a *p*-value of gene significance in VPA resistance less than 0.05 were recognized as significant module genes, and 562 genes were screened out accordingly for subsequent analysis (Supplementary Table S1).

### Identification of DEGs

A total of 70 genes were found to be differentially expressed between VPA-responsive and VPA-resistant patients (Supplementary Table S2). Among these DEGs, 47 were downregulated and 23 were upregulated. There were 26 genes identified to be differentially expressed between patients with naïve epilepsy and healthy control samples. Among these DEGs, 15 were downregulated and 11 were upregulated (Supplementary Table S3). A total of 74 genes were differentially expressed between patients with naive epilepsy and patients taking VPA. Among these DEGs, 28 were downregulated and 46 were upregulated (Supplementary Table S4).

### Identification of target gene and bio-function analysis

**Figure 6:**
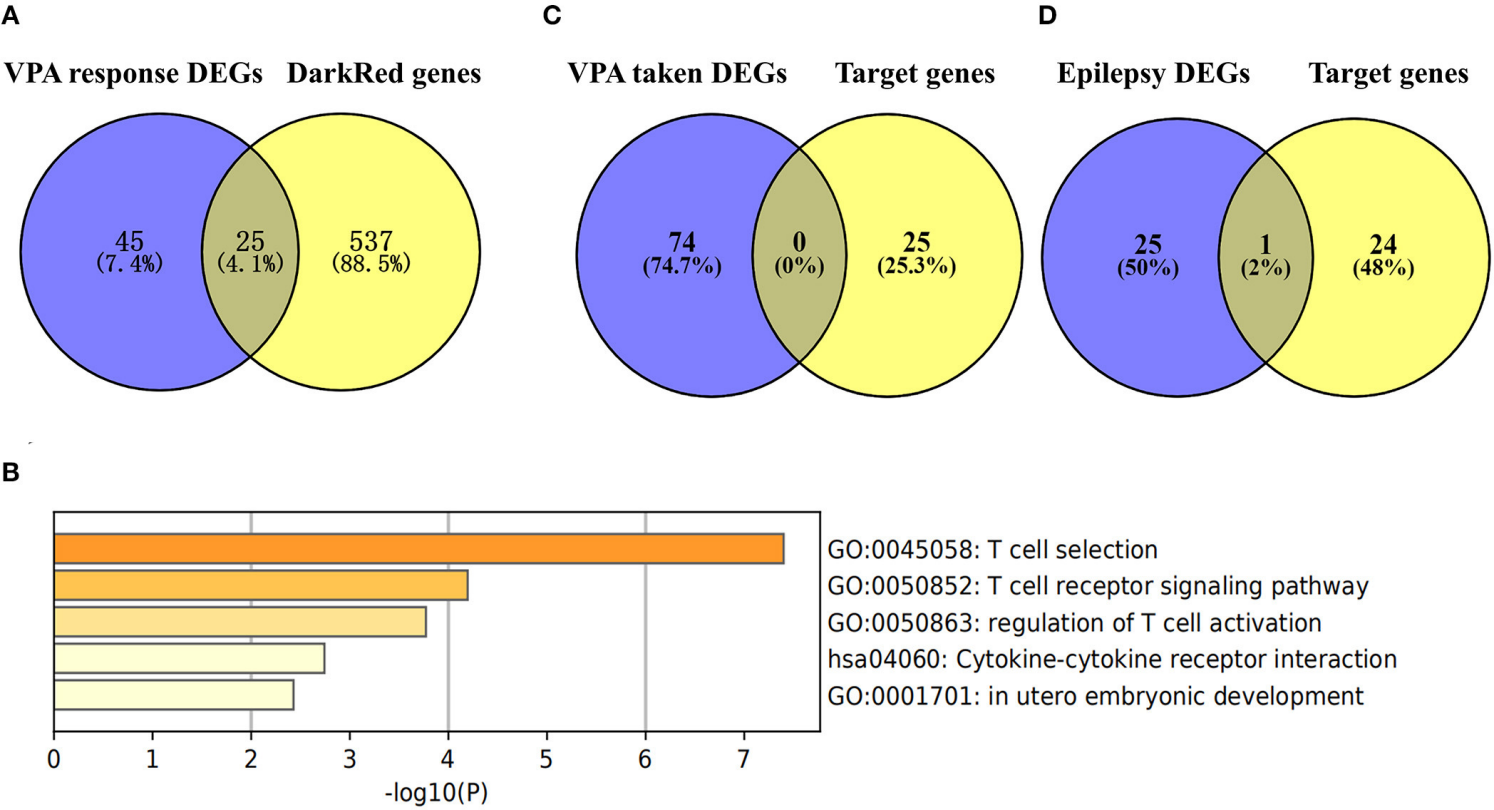
Identification of target genes in the key module and bio-function analysis. **(A)** Common genes of DEGs in VPA resistance (the blue circle) and the significant genes in the darkred module (the yellow circle). The DEGs in VPA resistance were obtained using the “Limma” algorithm by |Log2 FC| > 0.5 and *p-*value < 0.05. The darkred module was most related to VPA resistance through WGCNA analysis and the significant module genes were those with *p-*value of gene significance in VPA resistance less than 0.05. By comparing the genes, a total of 25 target genes were obtained. **(B)** The function of the 25 target genes was analyzed by Metascape and top 5 clustered terms were displayed. **(C)** Common genes of target genes and VPA taken DEGs (the blue circle). **(D)** Common genes of target genes and epilepsy DEGs (the blue circle) [“Log10(P)” is the *p*-value in log base 10. DEG, differentially expressed gene; VPA, valproate; WGCNA, weighted gene co-expression network analysis].

We used the Venn diagram to intersect the 562 significant genes in the darkred module with the 70 DEGs between VPA-responsive and VPA-resistant patients, and a total of 25 genes (*ATP23, C17orf97, CBS, CD3D, CD3G, CDR2, CRYZ, CXCR3, CXCR6, GATA3, GZMK, IL7R, IMPA1, LIME1, RBBP6, RPS26, RPS26P11, SF1, SIRPG, SNORA28, STMN3, THEMIS, TRAT1, ZNF683*, and *ZNF816*) were obtained (Figure 6A, Supplementary Table S5). Among the 25 target genes, 23 genes were upregulated in VPA-resistant patients except for *RBBP6* and *SNORA28*. Then, we analyzed the function of 25 target genes by Metascape. The enriched top 5 biological processes were T cell receptor recognition, T cell receptor signaling pathway, regulation of T cell activation, cytokine–cytokine receptor interaction, and *in utero* embryonic development (Figure 6B). Further functional analysis using the GeneCards human gene database showed that *CD3D, CD3G, CXCR3, CXCR6, GATA3, GZMK, IL7R, LIME1, SIRPG, THEMIS, TRAT1*, and *ZNF683* were directly involved in the T cell development, migration, and activation signaling pathway.

### Gene specificity verification

To test whether the 25 target genes associated with VPA resistance were affected by epilepsy or sodium VPA, we compared these 25 genes with the DEGs associated with taking VPA or the DEGs related to epilepsy. The results of the comparisons are presented in Figure 6C. From the Venn diagram, there was no intersection gene between the 25 target genes and DEGs associated with taking VPA. We also intersected these 25 target genes with the 26 DEGs associated with epilepsy and only one gene was common to both groups (Figure 6D).

## Discussion

Pharmacoresistance is an important unsolved problem in the field of epilepsy. Searching for hematological markers for epilepsy resistance is helpful to predict the drug response in patients with epilepsy, improve prognosis, and explore the potential mechanisms of drug-resistant epilepsy (20). WGCNA is a widely used bioinformatics analysis method that focuses on the gene modules involved in the common biological pathway and obtains the relationship between modules and traits in interest instead of several DEGs (13). In this article, we used WGCNA to obtain the gene module that most significantly correlated with VPA resistance; furthermore, we found the DEGs in this module closely participated in the T cell selection and activation pathway. Specifically, the DEGs in the significant module are associated with VPA resistance regardless of the presence or absence of seizure or VPA. As there was a close correlation between VPA resistance and epilepsy resistance (8), we proposed that the molecule expression of the T cell activation pathway in peripheral blood could be a good biomarker of epilepsy resistance.

Previous studies have also laid the foundation for T cell activation to be a biomarker of epilepsy resistance. Ouédraogo et al. identified that CD4+ T cells along with pro-inflammatory cytokines (e.g., interleukin-17A and tumor necrosis factor) expressed by CD4+ T cells were elevated in the peripheral blood of patients with drug-resistant epilepsy compared with that in patients with well-controlled epilepsy (21). T cells were upregulated not only in the peripheral blood of patients with intractable epilepsy but also in brain tissue. Tröscher et al. found that CD3+ as well as CD8+ T cell numbers were significantly elevated in the resected hippocampi of patients with temporal lobe epilepsy (TLE) compared with that in the healthy controls, although the numbers varied significantly among TLE subgroups (22). Animal models of intractable epilepsy also showed that T cells were increased in the epileptic hippocampus (23). Together, our study combined with the results of previous studies showed that the upregulation and activation of T cells in peripheral blood could be a good biomarker for drug-resistant epilepsy.

T cells could also participate in the physiological mechanism of epilepsy resistance. In TLE mice, 60–75% of T cells present in the hippocampus were cytotoxic CD8+ T cells, suggesting a potential role in the damage of hippocampal neurons that was regarded as an important pathophysiological basis for epileptogenesis of TLE (23). A further analysis of TLE showed that the number of T cells in the epileptic hippocampi was significantly correlated with the degree of neuronal loss, but not with seizure frequency or disease duration (22). Importantly, the attack of CD8+ T cells on hippocampal neurons was found to induce hippocampal sclerosis and TLE in limbic encephalitis (24). Pro-inflammatory CD4+ T cells could also cause neuronal damage *in vivo* (25) and directly lead to intractable seizure in an animal model of epilepsy partly through secreting interleukin-17A and granulocyte-macrophage colony-stimulating factor (26). The role of T cells in blood–brain barrier (BBB) injury has also attracted increasing attention (27). Multiple studies in neurological diseases such as Alzheimer's disease and multiple sclerosis suggested that T lymphocytes were closely related to BBB destruction (28, 29). Before entering the brain parenchyma, pro-inflammatory CD17+ T cells were interacted closely with vascular endothelial cells by promoting the downregulation of tight junction protein and adhesion protein, stimulating the expression of pro-inflammatory cytokines, and promoting the transmigration of CD4+ T cells (27, 28). The infiltrated CD8+ T cells also showed to be involved in BBB destruction through a perforin-dependent process (30). Removing T lymphocytes in peripheral blood by Fingomod could reduce BBB injury and P-glycoprotein expression (31). This evidence supported that peripheral blood-derived T cells might play an important role in BBB injury.

This study has some limitations. First, the sample size in our study is limited. Therefore, expanding the sample validation can improve the reliability of the research results. Second, further experimental verification of target gene expression in patients with epilepsy is necessary, and the undifferentiated T cell types need to be further explored in future studies. In addition, as a potential candidate biomarker, the sensitivity and specificity of T cell target gene expression in peripheral blood to predict epilepsy resistance need to be further validated in population data.

In summary, using WGCNA, our study has identified that peripheral blood T lymphocyte activation could be a good biomarker for VPA treatment response. Furthermore, our findings also have guiding significance for further revealing the pathophysiological mechanism of drug-resistant epilepsy.

## Data availability statement

The datasets presented in this study can be found in online repositories. The names of the repository/repositories and accession number(s) can be found below: https://www.ncbi.nlm.nih.gov/, GSE143272.

## Ethics statement

Ethical review and approval was not required for the study on human participants in accordance with the local legislation and institutional requirements. Written informed consent for participation was not required for this study in accordance with the national legislation and the institutional requirements.

## Author contributions

JM participated in bioinformatics analysis and drafting of the manuscript. QC participated in the research design, bioinformatics analysis, and manuscript revision. WW provided help with image production and the manuscript preparation. JZ participated in the research design. XL revised the manuscript. All authors read and approved the final manuscript.

## Conflict of interest

The authors declare that the research was conducted in the absence of any commercial or financial relationships that could be construed as a potential conflict of interest.

## Publisher's note

All claims expressed in this article are solely those of the authors and do not necessarily represent those of their affiliated organizations, or those of the publisher, the editors and the reviewers. Any product that may be evaluated in this article, or claim that may be made by its manufacturer, is not guaranteed or endorsed by the publisher.

## Abbreviations

VPA: valproate
WGCNA: weighted gene co-expression network analysis
DEGs: differentially expressed genes
BBB: blood brain barrier
TLE: temporal lobe epilepsy.
